# Excitonic Effects on the Ultrafast Nonlinear Optical Response of MoS_2_ and Fluorinated Graphene/MoS_2_ Heterostructure Films for Photonic Applications

**DOI:** 10.1021/acsami.4c16405

**Published:** 2024-11-08

**Authors:** Vasileios Arapakis, Michalis Stavrou, Georgios Skentzos, Dipak Maity, Tharangattu N. Narayanan, Stelios Couris

**Affiliations:** † Department of Physics, University of Patras, Patras 26504, Greece; ‡ Institute of Chemical Engineering Sciences (ICE-HT), Foundation for Research and Technology-Hellas (FORTH), Patras 26504, Patras, Greece; § Materials & Interface Engineering Laboratory, Tata Institute of Fundamental Research Hyderabad, Serilingampally Mandal, Hyderabad 500046, India

**Keywords:** MoS_2_, fluorographene, excitonic effects, *Z*-scan, optical Kerr effect

## Abstract

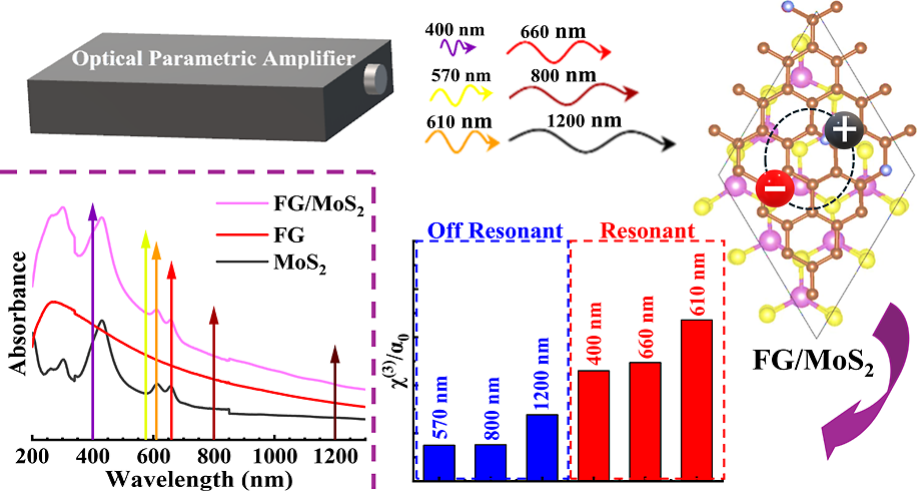

In the present work, the ultrafast nonlinear optical (NLO) response of some molybdenum disulfide (MoS_2_), fluorinated graphene (FG), and FG/MoS_2_ heterostructure thin films was studied using the *Z*-scan and optical Kerr effect techniques employing femtosecond laser pulses at different excitation wavelengths (i.e., 400, 570, 610, 660, 800, and 1200 nm). The experiments have shown that the NLO response of the MoS_2_ and MoS_2_/FG films was significantly enhanced when the films were excited with 400, 610, and 660 nm laser pulses due to resonance effects with the close-lying excitons in these nanostructures. For a better evaluation of the resonant enhancement of the NLO response, measurements were also carried out at off-resonant wavelengths, i.e., at 570, 800, and 1200 nm. The presence of excitons in the MoS_2_ and MoS_2_/FG films resulted in strong saturable absorption and self-defocusing, with exceptionally large values of third-order susceptibilities χ^(3)^ ranging from 10^–12^ to 10^–13^ esu. In addition, the NLO response of the MoS_2_/FG heterostructure was found to be stronger than that of the individual MoS_2_ and FG films, most probably attributed to interlayer carrier transfer. The determined NLO parameters of the studied nanostructures were found to be comparable to, and in some cases exceeded, those of other reported 2D materials known to exhibit a strong NLO response as well. These findings not only advance the fundamental understanding of the contributions of excitons on the NLO response/properties of transition metal dichalcogenide-based ultrathin films but also highlight the importance of excitons for tailoring their NLO response in view of various applications in advanced optoelectronics and photonic devices.

## Introduction

Two-dimensional (2D) layered nanostructures have currently become a subject of intense research due not only to their distinctive physical properties and the underlying physics^1−6^ but also to their potential use for a wide range of applications in electronics, photonics, and optoelectronics. While 2D materials were receiving increasing attention, significant advancements have been also made in designing heterostructures, consisting of two different 2D crystal materials stacked together via weak van der Waals forces, such as graphene derivatives and transition metal dichalcogenides (TMDs).^7−9^ Such heterostructures demonstrate important synergistic effects, which can be useful for practical applications. For instance, by combining the advantages of high carrier mobility of graphene with the tunable energy bandgap of a TMD, the fabrication of highly efficient field-effect transistors has become possible.^10^ Similarly, the combination of the semiconducting properties of TMDs with the high conductivity of graphene, realized in some TMD-graphene heterostructures, has enabled the development of nonvolatile memory cells.^11^ Moreover, other studies have shown that graphene-TMD heterostructures facilitate the formation of barrier-free contacts to TMD materials.^12,13^ In a more recent work, interfacial charge carrier dynamic studies have revealed that 2D heterostructures of fluorinated graphene (FG) and MoS_2_ exhibit superior optoelectronic properties, such as giant photoresponsivity, suitable for photodetectors’ and solar cells’ applications.^14,15^

Recently, research efforts have been focused on the investigation of the nonlinear optical (NLO) properties of these 2D heterostructures in view of their several potential applications in photonics and optoelectronics as saturable absorbers, optical limiters, optical switches, optical modulators, photodetectors, etc. It has been already reported that TMD-graphene heterostructures exhibit enhanced NLO response, attributed to interlayer charge transfer occurring from the MoS_2_ layer to the graphene one.^16^ On the other hand, the presence of excitons in TMDs affects significantly their optoelectronic properties, as, e.g., their absorption and emission characteristics, binding energies, valley polarization, photoconductivity, etc.^17,18^ So, it can be reasonably expected that the NLO response of TMD-graphene-based heterostructures can also be influenced by the presence of excitons. More specifically, excitons in TMDs can exhibit coherence effects when excited with fs laser pulses,^19^ which could lead to an enhanced NLO response by increasing the effective absorption cross-section, enabling resonant enhancement and quantum interference, and facilitating coherent emission.^20−24^

However, in most of the related literature research works concerning the ultrafast NLO response of such 2D nanostructures, the experimental investigations were performed around 800 nm (i.e., 1.55 eV), as this laser radiation is the most readily available among the various femtosecond laser sources. In order to investigate the effects of the presence of excitons on the NLO response of TMDs and TMD-graphene-based heterostructures, aiming for a better and more rational exploration of the potential of these materials in optoelectronics and photonics, the use of tunable fs laser radiation is necessary.^25^ In that view, the present work is the first attempt, to the best of our knowledge, to elucidate the effects of excitons’ presence on the ultrafast third-order NLO response (NLO absorption and refraction) of some MoS_2_ and FG/MoS_2_ heterostructure thin films, employing *Z*-scan and pump–probe optical Kerr effect (OKE) techniques. To that purpose, the output from an optical parametric amplifier (OPA) pumped by a fs Ti-sapphire laser system was used, and systematic measurements of the NLO response were performed at 400, 570, 610, 660, 800, and 1200 nm. Both studied nanostructures were found to exhibit a very strong NLO absorptive and refractive response, which was further enhanced when their excitons were resonantly excited.

## Experimental Section

### Material Synthesis and Characterization

The MoS_2_ and FG films were synthesized by chemical vapor deposition (CVD), as reported by some of the authors in their previous work.^14,15^ Large-area films were made by using the following methods: For the growth of monolayer MoS_2_, MoO_3_ powder was served as a Mo source and sulfur powder as a S source, while a fluorographite powder, with a F/C ratio of ∼0.6, was used as a source of carbon and fluorine for the growth of the large area FG film.^14^ The FG/MoS_2_ heterostructure films were synthesized using the CVD method by directly growing large-area uniform FG films on MoS_2_.^14,15^

The hybrid layer system (also individual layers), containing monolayer MoS_2_ [0.8 nm, atomic force microscopy (AFM) image, Figure S1a] and FG (∼4 nm, AFM image, Figure S1b), has a thickness of ∼5 nm. This hybrid layer grown on the SiO_2_/Si substrate is transferred to a quartz substrate (Techinstro TIQP 1001) using a single-step method. Polymethyl methacrylate (PMMA)-based polymer transfer method via spin coating the PMMA at 4000 rpm for 1 min and then heated at 90 °C for 10 min. These substrates were then put into a 2 M KOH solution for overnight. The separated films (MoS_2_/PMMA and FG/MoS_2_/PMMA hybrid films) from the SiO_2_ substrate were floated and scooped into the quartz substrates, followed by washing with acetone, isopropanol, and water. The optical images of such transferred films, as well as the Raman spectra and mapping indicating the presence of FG and MoS_2_ are shown in Figures S2–S4.

### NLO Measurements

#### *Z*-Scan Technique

**Figure 1:**
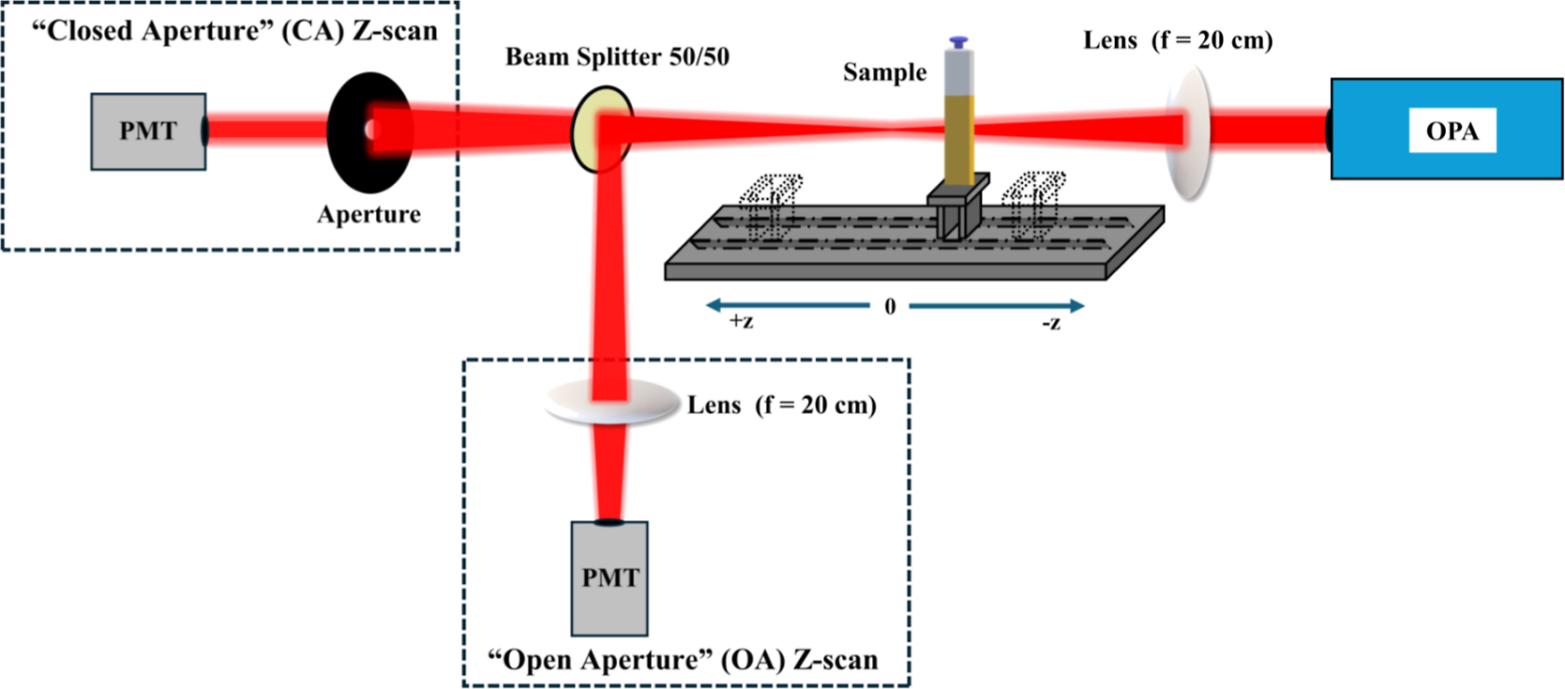
Schematic representation of the *Z*-Scan setup.

For the measurement of the third-order NLO response (i.e., NLO absorption and refraction) of the MoS_2_, FG, and FG/MoS_2_ heterostructure thin films, the *Z*-scan technique was employed.^26^ This technique allows for the simultaneous determination of the nonlinear absorption coefficient β (related to Imχ^(3)^) and the nonlinear refractive index parameter γ′ (related to Reχ^(3)^) from a single measurement. A schematic of the *Z*-scan experimental setup is shown in Figure 1. In brief, in this technique, the variation of the normalized transmittance of a sample exposed to variable laser intensity as it moves along the propagation direction (*z*-axis) of a focused laser beam, is simultaneously recorded by two different experimental configurations, namely, the “Open-Aperture” (OA) and “Closed-Aperture” (CA) *Z*-scans. In the former configuration, the laser beam transmitted through the sample is collected and measured, while in the latter configuration, only the central part of the transmitted laser beam is measured after it has passed through an aperture positioned in the far field, just in front of the detector. From the OA and CA *Z*-scan recordings, both the sign and the magnitude of the NLO absorption coefficient (β) and the nonlinear refractive parameter (γ′) of the sample can be determined.

In general, an OA *Z*-scan recording can exhibit a transmission maximum or minimum around the focal plane (i.e., at *z* = 0), indicating saturable absorption (SA) or reverse saturable absorption (RSA), corresponding to negative or positive NLO absorption coefficient β (<0 or >0), respectively. Likewise, in the case of negligible or small NLO absorption, the CA *Z*-scan can exhibit a prefocal transmission minimum followed by a postfocal maximum or vice versa, suggesting self-focusing (γ′ > 0) or self-defocusing (γ′ < 0) behavior, respectively. In the case of non-negligible NLO absorption, to account for its influence on the corresponding CA *Z*-scan, the latter was divided by the corresponding OA *Z*-scan, yielding the so-called “Divided” *Z*-scan.

Then, the magnitudes of the β and γ′, can be determined from the OA and the CA (or the “Divided”) *Z*-scan recordings through fitting with eqs 1 and 2, respectively^27^
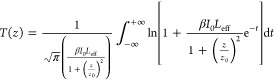
1
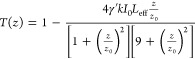
2where *T*(*z*) is the sample’s transmittance at each *z*-position, *z*_0_ is the Rayleigh length, *I*_0_ is the laser intensity at the focal plane, *L*_eff_ is the sample’s effective length, and *k* is the excitation wavenumber.

From the determined β and γ′, the imaginary, Imχ^(3)^, and real, Reχ^(3)^, parts of the third-order susceptibility χ^(3)^, can be deduced from eqs 3 and 4, respectively
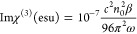
3
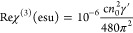
4where *c* is the speed of light, *n*_0_ is the linear refractive index, and ω is the frequency of the laser radiation.

### OKE Technique

**Figure 2:**
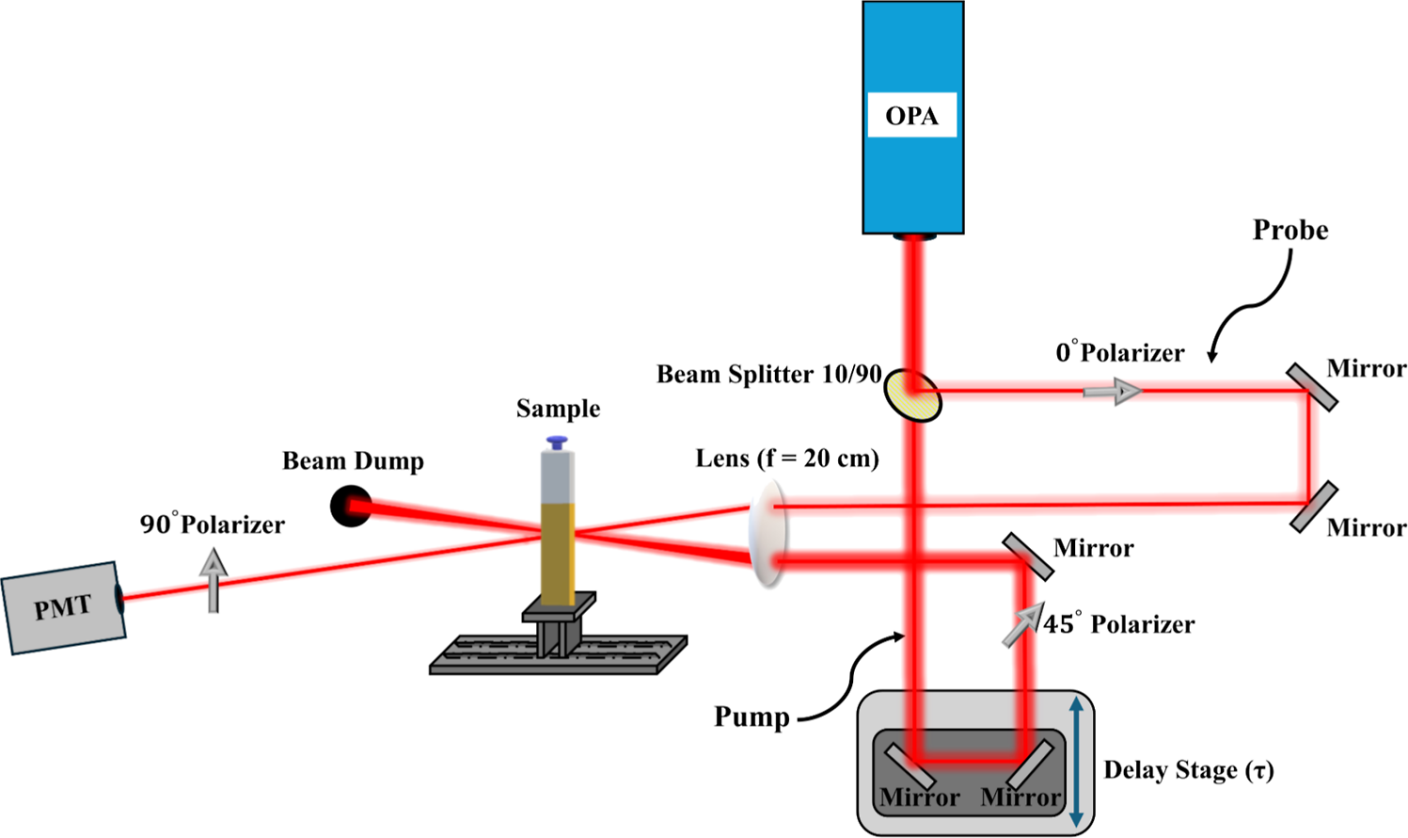
Schematic representation of the OKE experimental setup.

In addition to the *Z*-scan technique, the OKE technique was also used to determine the magnitude of the third-order susceptibility, Reχ^(3)^. OKE, being a pump probe technique, can be also used for the time-resolved study of the NLO response by inserting a controllable time delay between the pump and the probe beams.^27^ A schematic of the OKE experimental setup is presented in Figure 2. Briefly, in OKE, a laser beam is split into two parts, a strong (pump beam) and a weaker one (probe beam), having an intensity ratio of approximately 10:1. The pump beam induces a birefringence in the sample, while the probe beam experiencing this birefringence changes its polarization state (called the OKE signal). Then, the OKE signal is measured by a photodetector (e.g., a photomultiplier), and the magnitude of Reχ^(3)^ can be calculated, usually through comparison with a reference material, using the following relation
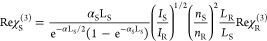
5where the subscripts S and R refer to the sample and reference materials, *I* is the OKE signal, n is the linear refractive index, α is the linear absorption coefficient (at the laser excitation wavelength), and *L* is the effective length of the sample.

### Laser Source

Both the *Z*-scan and OKE experiments were conducted using a CPA mode-locked Ti:sapphire laser system (Trident X, Amplitude Technologies) operating at 800 nm, delivering 70 fs laser pulses at a repetition rate of 10 Hz. For the experiments at other excitation wavelengths, both the second harmonic (SHG) of the fundamental laser beam and the output from an OPA (Palitra, Amplitude Technologies), pumped by the same Ti:sapphire laser, were used. So, laser light at 400, 570, 610, 660, and 1200 nm was produced, corresponding to photon energies of 3.1, 2.17, 2.03, 1.88, and 1.03 eV, respectively. The pulse duration of the laser pulses used, at each different excitation wavelength, was determined just in front of the samples by an autocorrelator and was found to be about 40, 60, 53, 62, and 80 fs, respectively.

The laser beam was focused onto the samples by means of a 20 cm focal length planoconvex quartz lens. The beam radius at the focus was determined using a CCD camera for each excitation wavelength. It was found to be approximately 20, 40, 48, 74, 25, and 35 μm at 400, 570, 610, 660, 800, and 1200 nm, respectively. From the knowledge of the laser pulse duration and beam width in each case, the corresponding laser intensity employed was calculated. The *Z*-scan and OKE measurements were performed for a wide range of incident laser intensities, ranging from 40 to 860 GW/cm^2^.

Τo account for any nonuniformity of the films, *Z*-scan and OKE measurements were performed at different places on the surface of the films and at different laser intensities. Then, the data were analyzed, and the NLO parameters were determined. This approach was applied for all studied samples, thus ensuring that the determined NLO parameters are accurate and realistically representative of the NLO response of the film. This methodology is schematically illustrated in Figure S5.

## Results and Discussion

**Figure 3:**
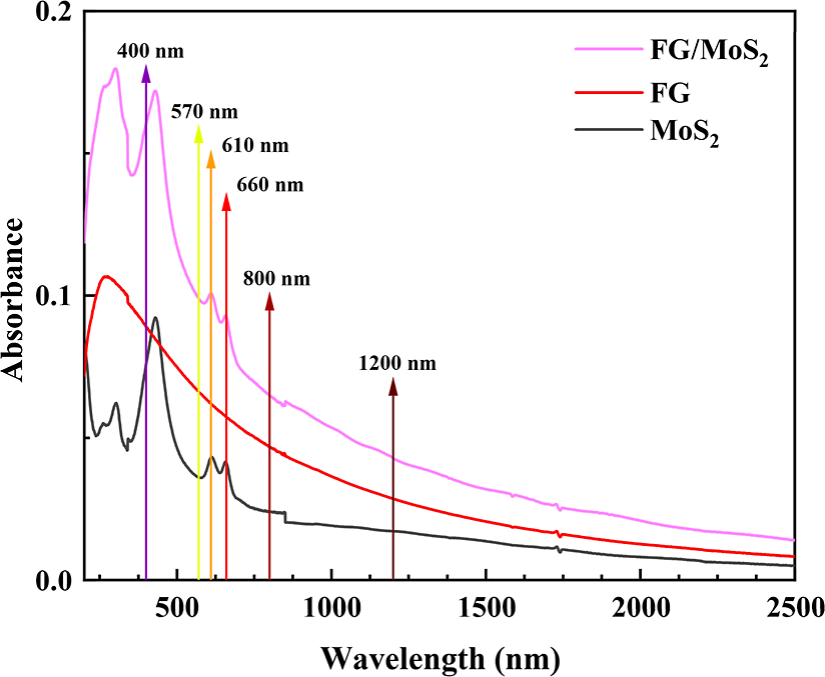
UV–vis–NIR absorption spectra of MoS_2_, FG, and FG/MoS_2_ thin films. The arrows represent the excitation wavelengths.

In Figure 3, the UV–vis–NIR absorption spectra of the MoS_2_, FG, and FG/MoS_2_ thin films obtained using a double beam UV–vis–NIR spectrophotometer (Jasco V-670) are shown after having been corrected for the absorption of the substrate. As shown, the MoS_2_ and FG/MoS_2_ films exhibited two distinct peaks at ∼610 nm (2.03 eV) and ∼660 nm (1.87 eV), along with a broad absorption band at ∼427 nm (2.9 eV). The former peaks correspond to direct excitonic transitions, namely the B and A excitons, from the spin–orbit split valence band to the conduction band at the *K* point of the Brillouin zone, while the band at 427 nm is assigned to excitonic transitions occurring between the high-density states in the valence band and the conduction band at the *M* point of the Brillouin zone.^28^ The UV–vis–NIR absorption spectrum of FG was rather structureless throughout the visible/NIR spectrum, exhibiting only an absorption maximum at ∼270 nm (4.59 eV), attributed to the π–π* transitions of the aromatic C=C bonds in the sp^2^-hybridized regions.^29^

From the optical absorption spectra, the values of the energy band gap (*E*_g_) of the MoS_2_, FG, and FG/MoS_2_ films were estimated using the Tauc plot method.^30^ Specifically, as can be seen from Figure S6a, the *E*_g_ value of the FG film, considering direct allowed optical transitions, was estimated to be ∼1.7 eV, but close to the value reported (1.2 eV) using ellipsometry-based analyses on similar films.^31^ This bandgap value is significantly lower than the reported one (i.e., 3 eV) for fully fluorinated graphene, due to the partial fluorination of the FG samples.^29^ Regarding the *E*_g_ value of the MoS_2_ sample, assuming direct allowed transitions, a value of ∼1.9 eV was estimated (see Figure S6b), in very good agreement with other reports for a MoS_2_ monolayer structure.^32^ Lastly, the bandgap of the FG/MoS_2_ heterostructure, presenting an indirect optical bandgap as confirmed by theoretical simulations,^15^ was estimated to be ∼1.4 eV (see Figure S6c).

**Figure 4:**
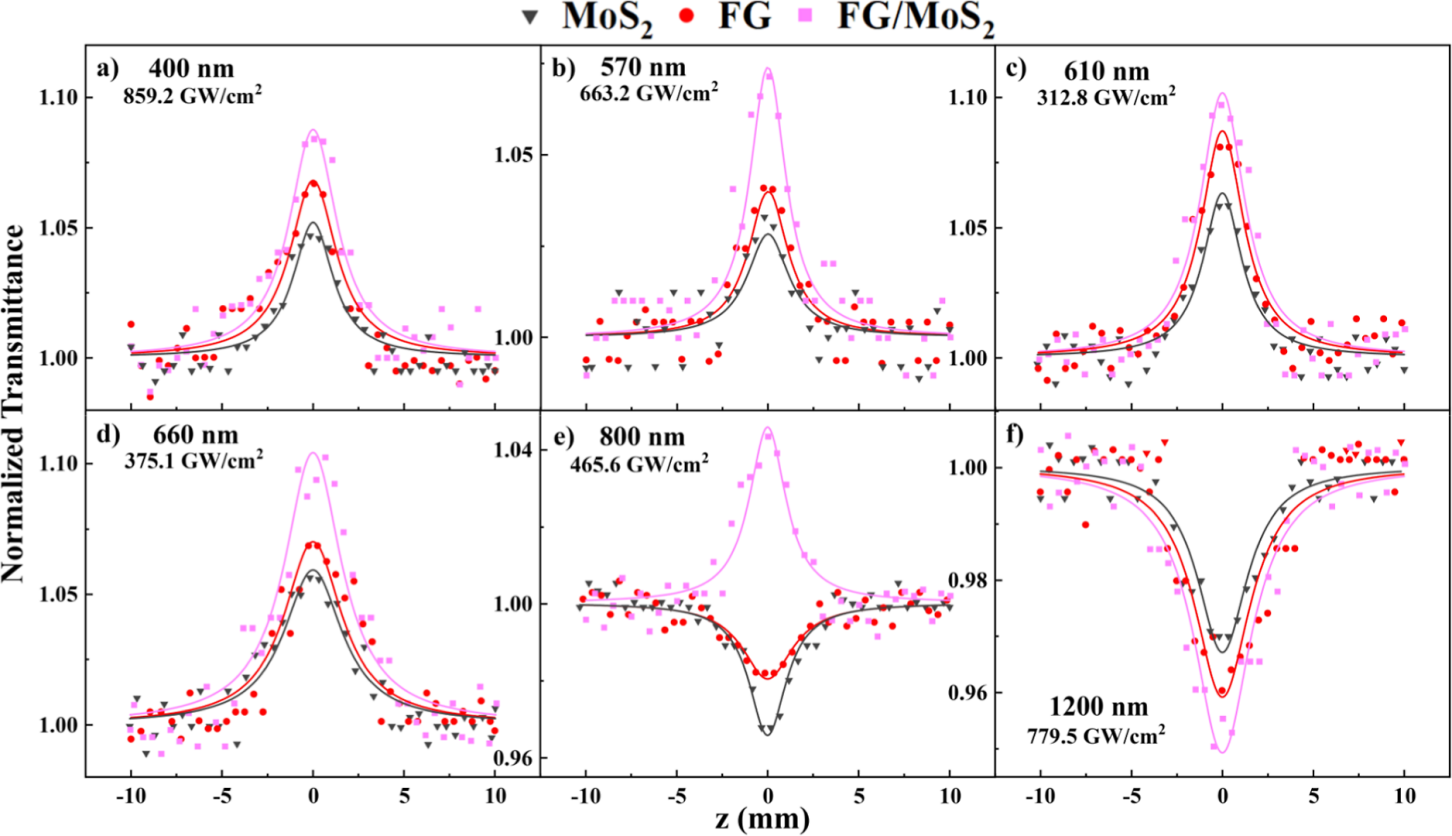
OA *Z*-scans of MoS_2_, FG and FG/MoS_2_ heterostructure thin films at (a) 400, (b) 570, (c) 610, (d) 660, (e) 800, and (f) 1200 nm.

In Figure 4, some representative OA *Z*-scans of the MoS_2_, FG, and FG/MoS_2_ thin films obtained using femtosecond laser excitation at various excitation wavelengths are shown. The solid points correspond to the experimental data points, while the solid lines correspond to the best fit in eq 1. The quartz substrate revealed significant NLO absorption at all excitation wavelengths, i.e., 400, 570, 610, 660, 800, and 1200 nm, for incident laser intensities exceeding 954, 860, 385, 412, 800, and 877 GW/cm^2^, respectively. For simplicity, the OA *Z*-scans, shown in Figure 4, were selected to correspond to laser intensities for which the substrate did not exhibit any measurable NLO absorption. So, from Figure 4a–d, it is apparent that all the samples presented a transmission maximum near the focal plane (i.e., at *z* = 0) under 400, 570, 610, and 660 nm laser excitation, denoting SA behavior. Similarly, the FG/MoS_2_ heterostructure exhibited SA behavior at 800 nm, while the MoS_2_ and FG films presented opposite behavior, i.e., RSA. Under 1200 nm excitation, all films exhibited an RSA behavior.

To better understand the SA/RSA behaviors of the studied nanostructures under the different excitation conditions used, the energy of the photons used for excitation in each case and the electronic structure of MoS_2_, FG and FG/MoS_2_ should be evoked. More specifically, the excitation of MoS_2_ and FG/MoS_2_ films by 400, 610, and 660 nm photons (see e.g., Figure 4a,c,d) is expected to be a quite efficient process, being an one-photon process (1PA) resonant with the excitons located at these spectral regions (e.g., see also Figure 3), leading to the formation of excitonic states lying within the bandgap.^17^ Similarly, excitation at 570 nm (∼2.2 eV) (see also Figure 4b), should be also an efficient one photon process (1PA) from the valence to the conduction band, considering the energy bandgap of MoS_2_ and FG/MoS_2_, being ∼1.9 and ∼1.4 eV, respectively. For the same reason, the excitations of the FG nanostructure by 400 nm (3.1 eV), 570 nm (2.17 eV), 610 nm (2.03 eV), and 660 nm (1.88 eV) photons (see also Figure 4a–d) are expected to be very efficient as well, considering the higher photon energies compared to the *E*_g_ value of FG, being ∼1.7 eV. The same situation holds in the case of the FG/MoS_2_ heterostructure when excited by 800 nm (1.55 eV) photons (see also Figure 4e), due to its lower energy bandgap, being 1.4 eV. In all the previous cases, the electronic excitations can occur readily, leading to the efficient depletion of the lower states, resulting in SA behavior, attributed to the Pauli exclusion principle. On the contrary, under 800 nm excitation, both the MoS_2_ and FG films exhibited a RSA (see Figure 4e). This situation is due to the lower photon energy than the respective bandgap energies, therefore requiring the absorption of two photons (2PA).^32^ Similarly occurs for all nanostructures under 1200 nm excitation, where the absorption of two photons is required as well (Figure 4f).

**Figure 5:**
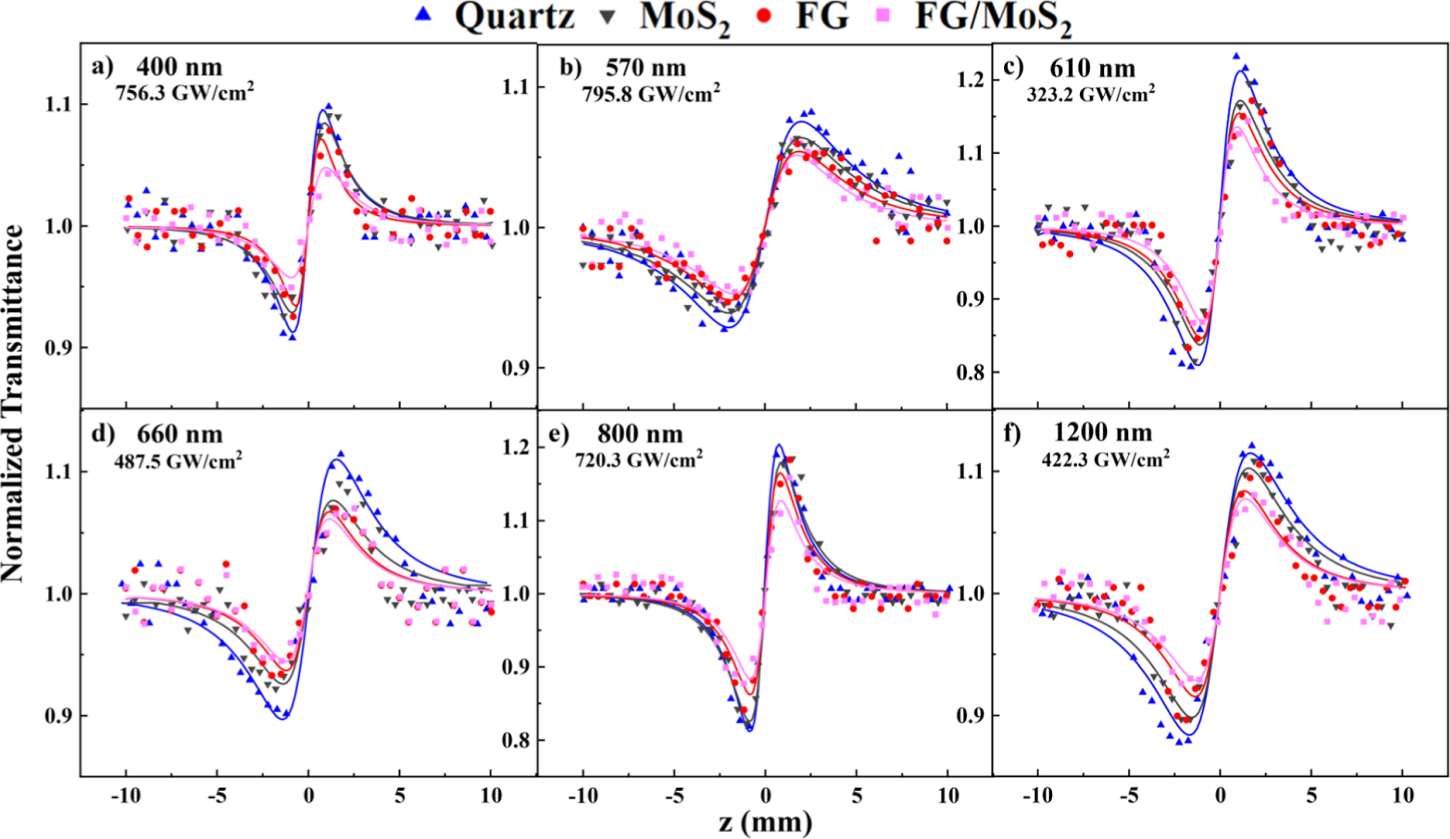
“Divided” *Z*-scans of MoS_2_, FG, and FG/MoS_2_ thin films at (a) 400, (b) 570, (c) 610, (d) 660, (e) 800, and (f) 1200 nm.

In Figure 5, some representative “divided” *Z*-scans of the MoS_2_, FG, and FG/MoS_2_ films and the quartz substrate, obtained under 400, 570, 610, 660, 800, and 1200 nm excitation, are presented. Again, the solid symbols correspond to the experimental data points, and the continuous lines represent the best fit of the experimental data points by eq 2. Since the present results have been obtained under ultrafast excitation conditions, the variation of the refractive index of the samples is mainly associated with the instantaneous bound-electronic response (i.e., Kerr-type nonlinearity).^33^ Therefore, it can be reasonably assumed that the NLO refractive response of the present samples can be primarily ascribed to their instantaneous bound-electronic response. As can be seen, all “divided” *Z*-scans presented a valley–peak transmittance configuration, suggesting self-focusing behavior (i.e., γ′ > 0), while the neat substrate exhibited Δ*Τ*_p–v_ values (i.e., the difference of the normalized transmittance between the peak and the valley of the “divided” *Z*-scan) larger than those of the films at all excitation wavelengths. A similar behavior was observed for all of the incident laser intensities used in the experiments, as shown in Figure S7. These findings indicate that all the films exhibit opposite sign refractive nonlinearity than that of the substrate, i.e., self-defocusing (i.e., γ′ < 0).

**Figure 6:**
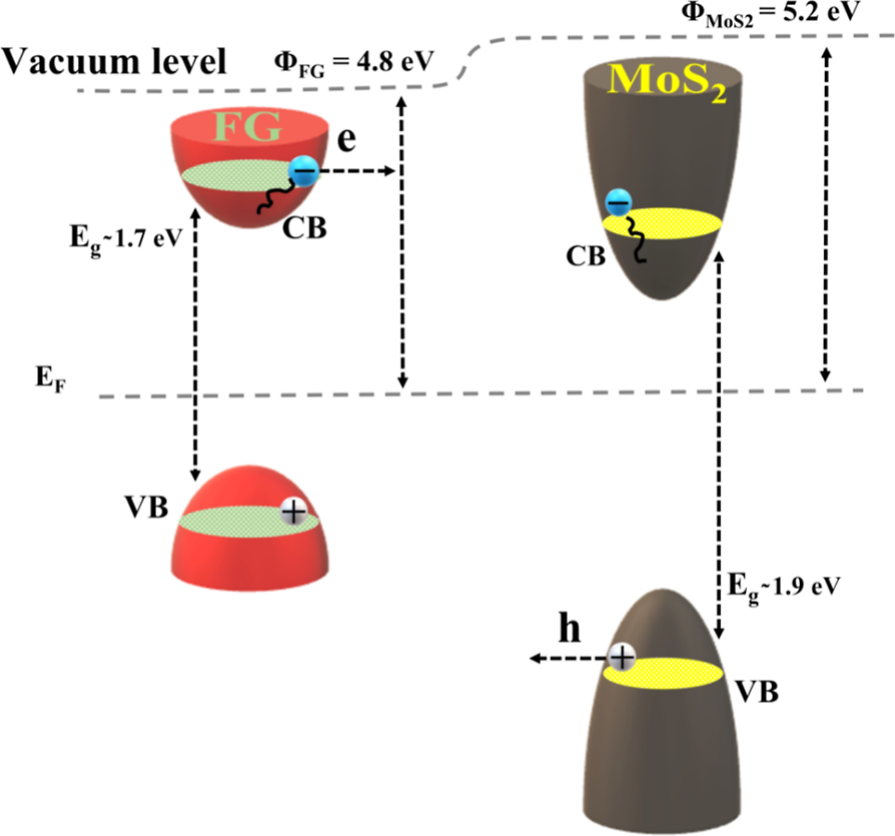
Schematic representation of charge transfer occurring between FG and MoS_2_. The band alignment was calculated using UV–vis absorption spectroscopy studies in ref (14).

From the analysis of the OA and “divided” *Z*-scans, the nonlinear absorption coefficient β, the nonlinear refractive index parameter γ′, the corresponding imaginary (Imχ^(3)^) and real (Reχ^(3)^) parts of the third-order susceptibility χ^(3)^, and the magnitude of the third-order susceptibility χ^(3)^, of the MoS_2_, FG, and FG/MoS_2_ nanostructures have been determined. They are summarized in Table 1. Because the determined NLO parameters depend on the absorption of the films, to make easier the comparisons between the samples and with other similar nanostructures, the corresponding figures of merit, FOM, have been also calculated, by normalizing the values of the NLO parameters with the corresponding absorption coefficient α_0_ at the laser excitation wavelength and have been included in Tables 1 and 2. As can be seen, the FG/MoS_2_ heterostructure revealed a significantly stronger NLO response than that of the individual MoS_2_ and FG samples, and their sum, at all excitation wavelengths examined, was attributed to the interactions between the MoS_2_ and FG layers occurring in the heterostructure films. To better understand this, the band structures of the MoS_2_ and FG samples, depicted schematically in Figure 6, should be considered.

**Table 1:** NLO Parameters of the MoS_2_, FG, and Heterostructure FG/MoS_2_ Films Determined by *Z*-Scan

excitation	sample	α_0_ (×10^4^ cm^–1^)	γ′ (×10^–14^ m^2^/W)	β (×10^–8^ m/W)	Reχ^(3)^ (×10^–9^ esu)	Imχ^(3)^ (×10^–9^ esu)	χ^(3)^ (×10^–9^ esu)	χ^(3)^/α_0_ (×10^–16^ esu·cm)
400 nm	Quartz	11.5 × 10^–6^	(23.9 ± 1.3) × 10^–7^	(12.8 ± 2.0) × 10^–7^	(35.9 ± 2.1) × 10^–7^	(4.0 ± 0.6) × 10^–7^	(36.2 ± 2.1) × 10^–7^	314 ± 18
	MoS_2_	253.3	–20.9 ± 1.15	–13.8 ± 1.88	–31.4 ± 1.73	–4.3 ± 0.58	31.7 ± 1.82	125 ± 7
	FG	43.2	–17.0 ± 1.6	–16.0 ± 1.27	–25.5 ± 1.7	–4.9 ± 0.39	26.0 ± 1.8	602 ± 41
	FG/MoS_2_	73.7	–44.6 ± 1.2	–36.4 ± 0.8	–67.1 ± 1.8	–11.2 ± 0.25	68.0 ± 1.82	922 ± 24
570 nm	Quartz	11.5 × 10^–6^	(11.8 ± 0.2) × 10^–7^	(9.3 ± 0.3) × 10^–7^	(17.3 ± 0.5) × 10^–7^	(4.0 ± 0.2) × 10^–7^	(17.8 ± 0.1) × 10^–7^	154 ± 1
	MoS_2_	103.3	–2.1 ± 0.2	–6.5 ± 0.8	–3.2 ± 0.3	–2.8 ± 0.3	4.2 ± 0.5	41 ± 5
	FG	37.4	–1.7 ± 0.1	–20.1 ± 0.6	–2.6 ± 0.1	–8.9 ± 0.3	9.3 ± 0.3	248 ± 8
	FG/MoS_2_	45.1	–6.2 ± 0.2	–21.8 ± 1.1	–9.9 ± 0.3	–9.3 ± 0.5	13.6 ± 0.6	301 ± 13
610 nm	Quartz	11.5 × 10^–6^	(14.5 ± 0.8) × 10^–7^	(12.0 ± 2.0) × 10^–7^	(21.2 ± 1.2) × 10^–7^	(5.1 ± 1.2) × 10^–7^	(21.8 ± 1.7) × 10^–7^	190 ± 15
	MoS_2_	123.8	–6.9 ± 0.2	–38.8 ± 3.3	–10.4 ± 0.3	–17.8 ± 1.9	20.6 ± 2.0	166 ± 16
	FG	35.7	–3.5 ± 0.3	–9.4 ± 0.5	–5.3 ± 0.4	–4.3 ± 0.2	6.8 ± 0.5	190 ± 14
	FG/MoS_2_	46.1	–29.2 ± 0.1	–95.4 ± 3.2	–43.9 ± 0.1	–43.7 ± 1.8	61.9 ± 1.9	1343 ± 41
660 nm	Quartz	11.5 × 10^–6^	(12.2 ± 0.5) × 10^–7^	(11.3 ± 0.5) × 10^–7^	(17.5 ± 0.8) × 10^–7^	(5.6 ± 0.3) × 10^–7^	(18.3 ± 0.8) × 10^–7^	159 ± 7
	MoS_2_	120.9	–1.3 ± 0.3	–29.7 ± 0.2	–2.0 ± 0.5	–18.0 ± 0.1	18.1 ± 0.5	149 ± 4
	FG	33.4	–0.5 ± 0.1	–13.6 ± 1.3	–0.7 ± 0.1	–6.8 ± 0.6	6.8 ± 0.5	204 ± 15
	FG/MoS_2_	42.4	–3.0 ± 0.1	–83.6 ± 1.6	–4.5 ± 0.1	–41.6 ± 1.0	41.9 ± 1.0	988 ± 23
800 nm	Quartz	11.5 × 10^–6^	(15.9 ± 0.7) × 10^–7^	(4.1 ± 0.9) × 10^–7^	(24.0 ± 1.0) × 10^–7^	(2.4 ± 0.6) × 10^–7^	(24.1 ± 1.0) × 10^–7^	210 ± 9
	MoS_2_	69.1	–0.20 ± 0.05	7.0 ± 0.5	–0.30 ± 0.08	4.1 ± 0.3	4.1 ± 0.3	59 ± 4
	FG	24.2	–2.6 ± 0.2	0.45 ± 0.06	–3.9 ± 0.4	0.3 ± 0.04	3.9 ± 0.4	161 ± 16
	FG/MoS_2_	29.5	–4.1 ± 0.1	–11.1 ± 1.0	–6.2 ± 0.2	–6.6 ± 0.9	9.0 ± 0.9	305 ± 30
1200 nm	Quartz	11.5 × 10^–6^	(16.1 ± 0.3) × 10^–7^	(5.2 ± 0.8) × 10^–7^	(24.2 ± 0.4) × 10^–7^	(4.7 ± 0.7) × 10^–7^	(24.7 ± 0.4) × 10^–7^	215 ± 7
	MoS_2_	48.9	–0.6 ± 0.1	3.7 ± 0.2	–0.9 ± 0.1	3.3 ± 0.2	3.4 ± 0.2	70 ± 4
	FG	16.1	–1.7 ± 0.3	2.2 ± 0.6	–2.5 ± 0.4	2.0 ± 0.5	3.2 ± 0.7	198 ± 43
	FG/MoS_2_	19.8	–4.7 ± 0.6	9.4 ± 0.7	–7.1 ± 0.9	8.5 ± 0.6	11.0 ± 1.1	555 ± 57

**Table 2:** Comparison of the NLO Response of MoS_2_, FG and FG/MoS_2_ Films with Other 2D Nanostructures Obtained under fs Laser Excitation Conditions

sample	excitation	α_0_ (×10^4^ cm^–1^)	γ′ (×10^–14^ m^2^/W)	β (×10^–8^ m/W)	Reχ^(3)^ (×10^–9^ esu)	Imχ^(3)^ (×10^–9^ esu)	Reχ^(3)^/α_0_ (×10^–16^ esu·cm)	Imχ^(3)^/α_0_ (×10^–16^ esu·cm)	ref
MoS_2_	400 nm, 50 fs	253.3	–20.9	–13.8	–31.4	–4.3	124	17	this work
	800 nm, 70 fs	69.1	–0.2	7.0	–0.3	4.1	4	59	
FG	400 nm, 50 fs	43.2	–17.0	–16.0	–25.5	–4.9	590	113	
	800 nm, 70 fs	24.2	–2.6	0.45	–3.9	0.27	161	11	
FG/MoS_2_	400 nm, 50 fs	73.7	–44.6	–36.4	–67.1	–11.2	910	152	
	800 nm, 70 fs	29.5	–4.1	11.1	–6.2	6.6	210	224	
graphene/ODCB	800 nm, 50 fs	5.7 × 10^–4^	7.1 × 10^–7^		10.7 × 10^–7^		1.9		(36)
graphene/CHP	800 nm, 100 fs	17.85 × 10^–4^		–152 × 10^–7^		–87 × 10^–7^		4.9	(37)
graphene/glass	800 nm, 35 fs	2.06		–116 × 10^–3^		–0.28		136	(39)
MoS_2_/NMP	800 nm, 100 fs	2.37 × 10^–4^		–46 × 10^–7^		–25.2 × 10^–7^		10.6	(38)
MoS_2_/NVP		1.66 × 10^–4^		–17.8 × 10^–7^		–10.3 × 10^–7^		6.2	
MoS_2_/CHP		2.85 × 10^–4^		–58 × 10^–7^		–33 × 10^–7^		11.6	
WS_2_/glass	800 nm, 35 fs	2.18		–76 × 10^–3^		–0.14		64.2	(39)
BP/NMP	800 nm, 100 fs	3.86 × 10^–4^	–(9.37–20.7) × 10^–7^	–40.8 × 10^–7^	–(13.6–30.1) × 10^–7^	–22.3 × 10^–7^	3.5–7.8	26.3	(40,41)
BP/IPA	400 nm, 100 fs	3.49 × 10^–4^		–162 × 10^–7^		–38.6 × 10^–7^		11.1	(40)
Ti_3_C_2_T_*x*_/glass	800 nm, 95 fs	120 × 10^–4^	–46.6 × 10^–7^	–117 × 10^–7^	–474 × 10^–7^	–68.5 × 10^–7^	3.9	0.6	(42)
	1064 nm, 95 fs		–34.7 × 10^–7^	–38.6 × 10^–7^	–353 × 10^–7^	–29.6 × 10^–7^	2.9	0.25	
SiNS-H	400 nm, 50 fs	2.55 × 10^–4^	4.9 × 10^–7^	–5.6 × 10^–7^	7 × 10^–7^	–1.5 × 10^–7^	2.75	0.6	(43)
SiNS-H	800 nm, 70 fs		22 × 10^–7^		32 × 10^–7^		12.5		
hematene	400 nm, 50 fs	2.5 × 10^–4^		–33.2 × 10^–7^		–7.5 × 10^–7^		3	(44)
magnetene				–21.4 × 10^–7^		–4.8 × 10^–7^		1.92	
MoS_2_/graphene	800 nm, 70 fs	5.4				3.2		592	(16)
ZnTTBPc/rGO	540 nm, 100 fs	2.87 × 10^–4^		23 × 10^–5^				429.4	(45)

So, given that MoS_2_ is an n-type semiconductor,^34^ its Fermi level is closer to the conduction band. In contrast, due to the electron-withdrawing nature of FG (a p-doped semiconductor), its Fermi level is expected to be near the valence band. Upon excitation of this p–n heterojunction, photogenerated electrons in the conduction band of FG can be transferred to the corresponding conduction band of MoS_2_, considering the lower work function of FG than that of MoS_2_, resulting in the creation of an internal electric field from FG to MoS_2_.^35^ This results in the accumulation of negative charges in MoS_2_ and, correspondingly, positive charges in FG. In fact, the charge transfer occurring in these FG/MoS_2_ heterostructures has been experimentally confirmed by both photoluminescence and transient absorption measurements.^14^ Therefore, it can be reasonably assumed that carrier transfer between MoS_2_ and FG can result in enhancement of the NLO response of the FG/MoS_2_ heterostructure. In particular, under 400, 570, 610, 660, and 800 nm laser pulses, interlayer charge transfer can enhance the SA in FG/MoS_2_ by extending the lifetime of the excited states in the heterostructure compared to that in individual FG and MoS_2_. On the other hand, under 1200 nm laser excitation, where the 2PA process occurs, charge transfer is likely not so important to enhance the NLO absorption. Instead, the formation of the heterostructure, which leads to lower energy bandgaps, assists resonant excitation through 2PA with low-energy infrared photons. Concerning the enhanced NLO refraction in the FG/MoS_2_ heterostructure, it is attributed to charge transfer, which modifies the electronic distribution and creates a strong dipole moment. The latter can enhance material’s ability to be polarized in response to an external electric field, resulting in a significant variation in the nonlinear refractive index.

**Figure 7:**
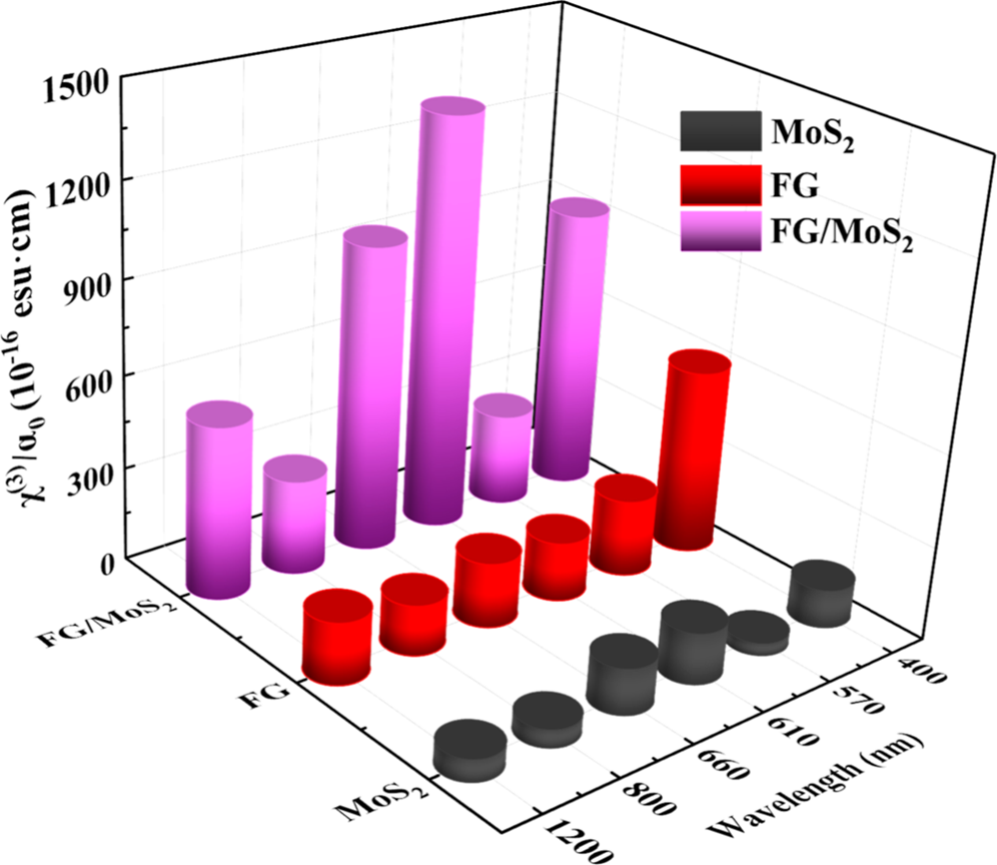
Variation of the χ^(3)^/α_0_ values of MoS_2_, FG, and FG/MoS_2_ films under 400, 570, 610, 660, 800, and 1200 laser excitation.

In Figure 7, the determined FOM χ^(3)^ values, i.e., χ^(3)^/α_0_, of MoS_2_, FG, and FG/MoS_2_ at the different laser excitation wavelengths are presented. As shown, the FOM values of MoS_2_ and FG/MoS_2_ exhibit a significant enhancement under 400, 610, and 660 nm excitation, attributed to the resonance excitation of their respective excitonic states present in these spectral regions (see, e.g., the UV–vis–NIR absorption spectra of Figure 3). On the other hand, FG does not exhibit similar enhancement due to the absence of such an excitonic structure.

For comparison purposes, the NLO parameters of some other van der Waals (vdW) 2D materials [e.g., graphene, TMDs (MoS_2_, WS_2_, and MoSe_2_), black phosphorus (BP), MXenes (Ti_3_C_2_Tx), and hydride-terminated silicon nanosheets (SiNS-H)],^36−43^ some non-vdW 2D materials (e.g., hematene and magnetene)^44^ and some other 2D heterostructures [e.g., MoS_2_/Graphene and zinc tetra *tert*-butyl phthalocyanine/reduced graphene oxide (ZnTTBPc/rGO) nanocomposites],^16,45^ all reported to exhibit strong NLO response, are included in Table 2. As can be seen from their FOM values, the studied MoS_2_, FG, and FG/MoS_2_ samples exhibit comparable, and in some cases, even stronger, NLO absorption and refraction than the other nanostructures. From this table, particular emphasis should be given to the FG/MoS_2_ heterostructure, which exhibits superior NLO absorption and refraction properties compared with most of the other 2D nanostructures listed. These findings position the FG/MoS_2_ heterostructure among the most promising NLO materials currently known, with potential applications spanning a wide range of fields, including laser technologies (e.g., saturable absorbers), optical protection (e.g., limiters), telecommunications and optical/quantum computing (e.g., switches and modulators), medical technologies, and more.

Since the studied films exhibited strong SA responses at 400, 570, 610, 660, and 800 nm (due to excitonic effects and interlayer charge transfer), the saturable laser intensity *I*_sat_ and the modulation depth α_s_ are of great importance for mode-locking and *Q*-switching applications. As shown in Figure S8 and Table S1, MoS_2_ and FG/MoS_2_ exhibit excellent SA properties, characterized by low saturable intensities and high modulation depths, particularly for the wavelengths at which their excitons are excited. These properties are comparable to or significantly better than those of other 2D materials reported for their strong SA, including graphene, TMDs like MoS_2_, WS_2_, and MoSe_2_, BP, MXenes (e.g., Ti_3_C_2_T_*x*_), silicon nanosheets (SiNSs), and non-van der Waals 2D materials such as hematene and magnetene.^37−40,42−44^ Furthermore, for the films that exhibited RSA behavior due to TPA under 800 and 1200 nm laser pulses, their optical limiting efficiency was assessed by determining the optical limiting onset (OL_on_), defined as the laser fluence where the sample’s transmittance begins to deviate from the Beer–Lambert law. As shown in Figure S8, the studied films exhibited OL_on_ values of the order of mJ/cm^2^. Notably, the FG/MoS_2_ heterostructure demonstrated lower OL_on_ values compared to the individual FG and MoS_2_ materials under 1200 nm laser pulses, indicating more efficient optical limiting, most likely due to more efficient interlayer charge transfer. Importantly, these values are comparable to or significantly lower than those reported for various 2D materials.^46−49^ These findings suggest that depending on the excitation conditions, the studied films could be effectively used as saturable absorbers for laser pulse generation or as optical limiters to protect sensitive sensors from high-power laser exposure.

**Figure 8:**
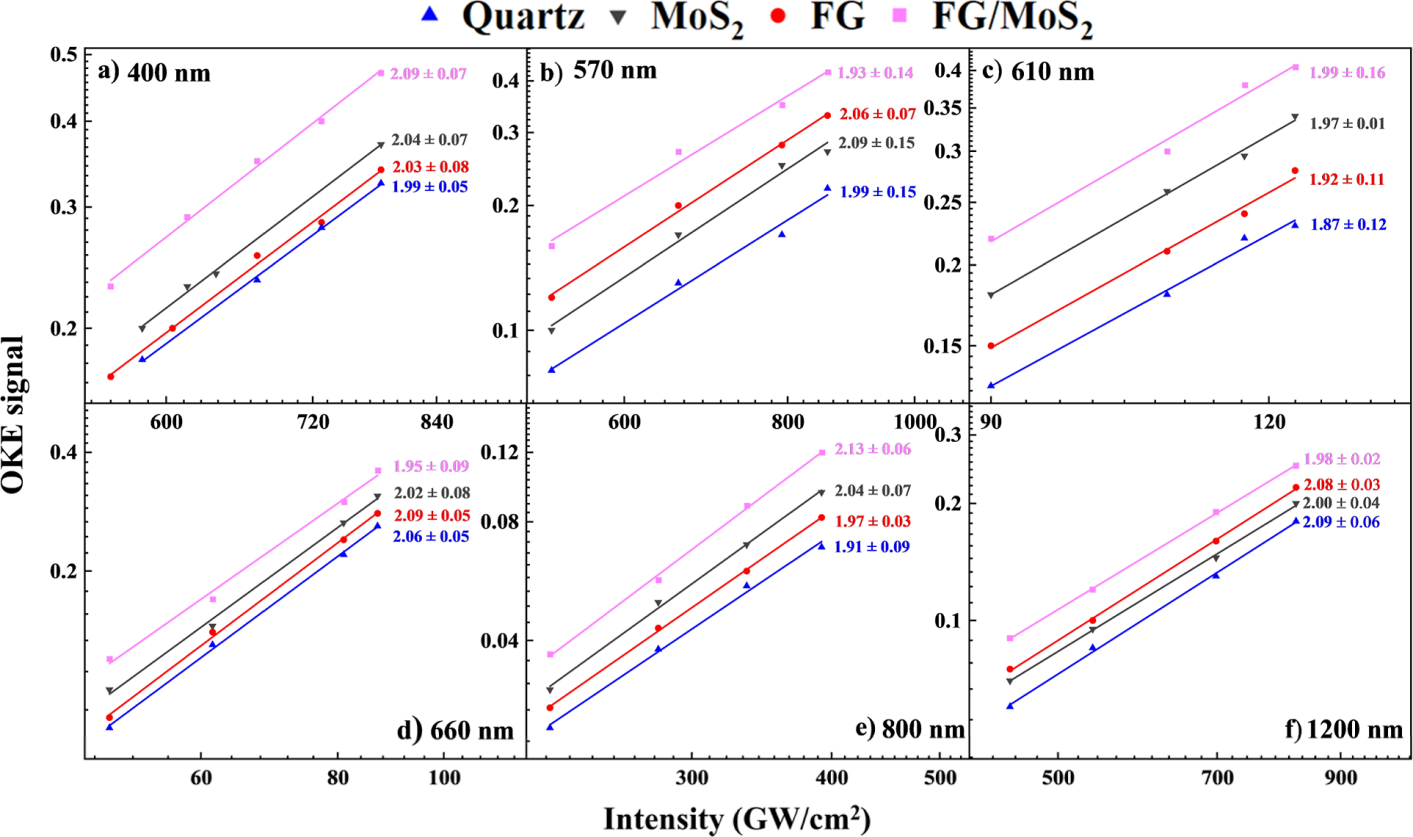
Dependence of the OKE signal on pump beam intensity at (a) 400 nm, (b) 570 nm, (c) 610 nm, (d) 660 nm, (e) 800 nm, and (f) 1200 nm.

Next, the results concerning the NLO response of the MoS_2_, FG, and FG/MoS_2_ films obtained by the OKE technique are presented and discussed. As in the case of the *Z*-scan investigation, the OKE experiments were performed under 400, 570, 610, 660, 800, and 1200 nm fs laser excitation. The intensity of the pump beam was varied within an appropriate range (different for each nanostructure to avoid saturation phenomena, etc.), while the intensity of the probe beam was kept constant at 78, 88, 13, 10, 40, and 80 GW/cm^2^ at 400, 570, 610, 660, 800, and 1200 nm, respectively. In all cases, the pump and probe beam intensities used were carefully chosen to ensure a good signal-to-noise ratio. In Figure 8, the variation of the OKE signal with the pump beam intensity is shown for the different nanostructures studied. In all cases, the OKE signal was found to exhibit a quadratic dependence on the pump beam intensity, consistent with third-order optical nonlinearity according to theoretical assumptions.^50^

For the determination of the χ^(3)^ of the films, the quartz substrate was used as a reference. The χ^(3)^ values of the substrate used as reference at each excitation wavelength, were those determined previously by *Z*-scan; they were (36.2 ± 2.1) × 10^–16^, (17.8 ± 0.1) × 10^–16^, (21.8 ± 1.7) × 10^–16^, (17.3 ± 0.8) × 10^–16^, (24.1 ± 1.0) × 10^–16^, and (24.7 ± 0.4) × 10^–16^ esu at 400, 570, 610, 660, 800, and 1200 nm, respectively. The determined χ^(3)^ values of the quartz substrate under 400 and 800 nm laser excitation are in excellent agreement with values reported in the literature.^51,52^ However, reference values for the other excitation wavelengths (i.e., 570, 610, 660, and 1200 nm) do not exist in the literature. The determined χ^(3)^ values of the films were then deduced using eq 5, and they are shown in Table 3.

**Table 3:** NLO Parameters of MoS_2_, FG, and FG/MoS_2_ Thin Films Obtained through ΟΚΕ Measurements

excitation	sample	α_0_ (×10^4^ cm^–1^)	|Reχ^(3)^| (×10^–9^ esu)	|Reχ^(3)^|/α_0_ (×10^–16^ esu·cm)
400 nm	Quartz	11.5 × 10^–6^	(36.2 ± 2.1) × 10^–7^	313 ± 18
	MoS_2_	253.3	39.9 ± 0.7	157 ± 3
	FG	43.2	36.2 ± 0.7	838 ± 16
	FG/MoS_2_	73.7	54.7 ± 1.8	742 ± 24
570 nm	Quartz	11.5 × 10^–6^	(17.8 ± 0.1) × 10^–7^	147 ± 4
	MoS_2_	103.3	2.6 ± 0.1	25 ± 1
	FG	37.4	2.8 ± 0.2	74 ± 5
	FG/MoS_2_	45.1	6.6 ± 0.1	145 ± 2
610 nm	Quartz	11.5 × 10^–6^	(21.8 ± 1.7) × 10^–7^	182 ± 13
	MoS_2_	123.8	15.1 ± 0.2	122 ± 2
	FG	35.7	3.3 ± 0.1	92 ± 2
	FG/MoS_2_	46.1	33.0 ± 0.1	716 ± 2
660 nm	Quartz	11.5 × 10^–6^	(17.3 ± 0.8) × 10^–7^	147 ± 7
	MoS_2_	120.9	2.5 ± 0.1	21 ± 1
	FG	33.4	0.5 ± 0.01	15 ± 3
	FG/MoS_2_	42.4	3.6 ± 0.1	85 ± 2
800 nm	Quartz	11.5 × 10^–6^	(24.1 ± 1.0) × 10^–7^	209 ± 9
	MoS_2_	69.1	3.4 ± 0.2	49 ± 3
	FG	24.2	3.2 ± 0.3	132 ± 12
	FG/MoS_2_	29.5	6.4 ± 0.4	217 ± 14
1200 nm	Quartz	11.5 × 10^–6^	(24.7 ± 0.4) × 10^–7^	209 ± 3
	MoS_2_	48.9	1.1 ± 0.1	23 ± 2
	FG	16.1	1.8 ± 0.1	111 ± 6
	FG/MoS_2_	19.8	5.0 ± 0.1	252 ± 5

**Figure 9:**
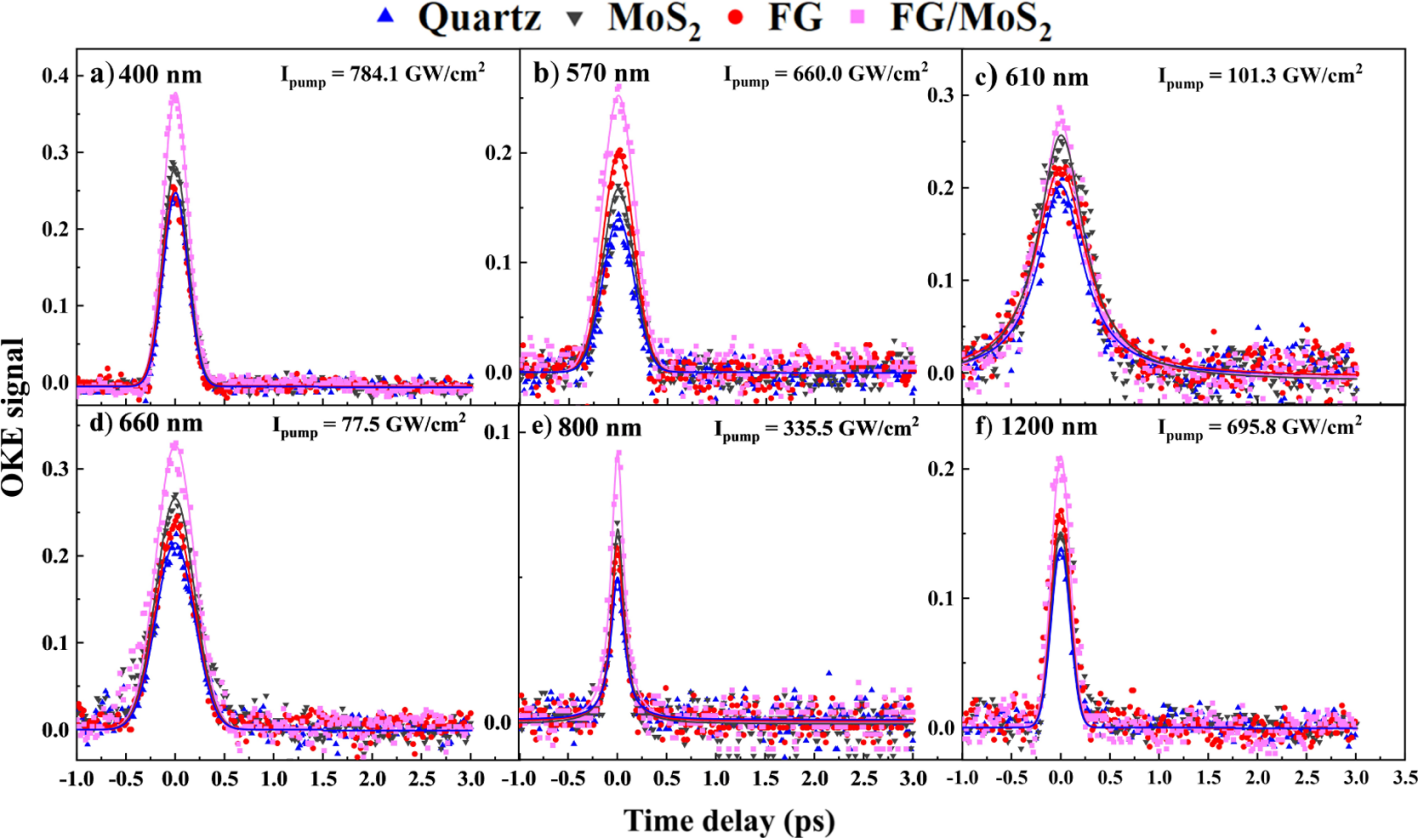
OKE signal variation with the delay time between the pump and probe beams, at (a) 400, (b) 570, (c) 610, (d) 660, (e) 800, and (f) 1200 nm.

In Figure 9, the variation of the OKE signal with the time delay between the pump and the probe beams for the studied films and the quartz substrate is presented. The experimental conditions, i.e., laser excitation wavelength and pump beam intensity, of each experiment are shown in the corresponding figure. The solid symbols correspond to the experimental data points, and the continuous lines correspond to the fitting of the experimental data points with a Gaussian function. In fact, the continuous lines represent the laser autocorrelation profile and imply an electronic origin NLO refractive response. As can be seen, the variation of the OKE signal with the delay time between the pump and the probe laser beams was found to be quite symmetric around zero time delay for all excitation wavelengths and samples. The time width of the autocorrelation traces presented in this figure were determined to be ∼250, ∼320, ∼450, ∼390, ∼150, and ∼190 fs for the 400, 570, 610, 660, 800, and 1200 nm laser beams, respectively, i.e., much larger than the duration of the 50, 60, 53, 62, 70, and 80 fs pulses measured in front of the *Z*-scan setup. This temporal broadening of the laser pulse is attributed to dispersion effects and other nonlinear optical processes, as e.g., self-phase modulation, occurring during the propagation of the laser beam through the different optical components of the experimental setup and the substrate of the samples. It is noteworthy that the absence of carrier relaxation processes in the OKE signals of the films is most likely attributed to the fact that these processes occur on shorter time scale(s) than the pulse duration. Similar behaviors have also been reported elsewhere in the literature.^53^

**Figure 10:**
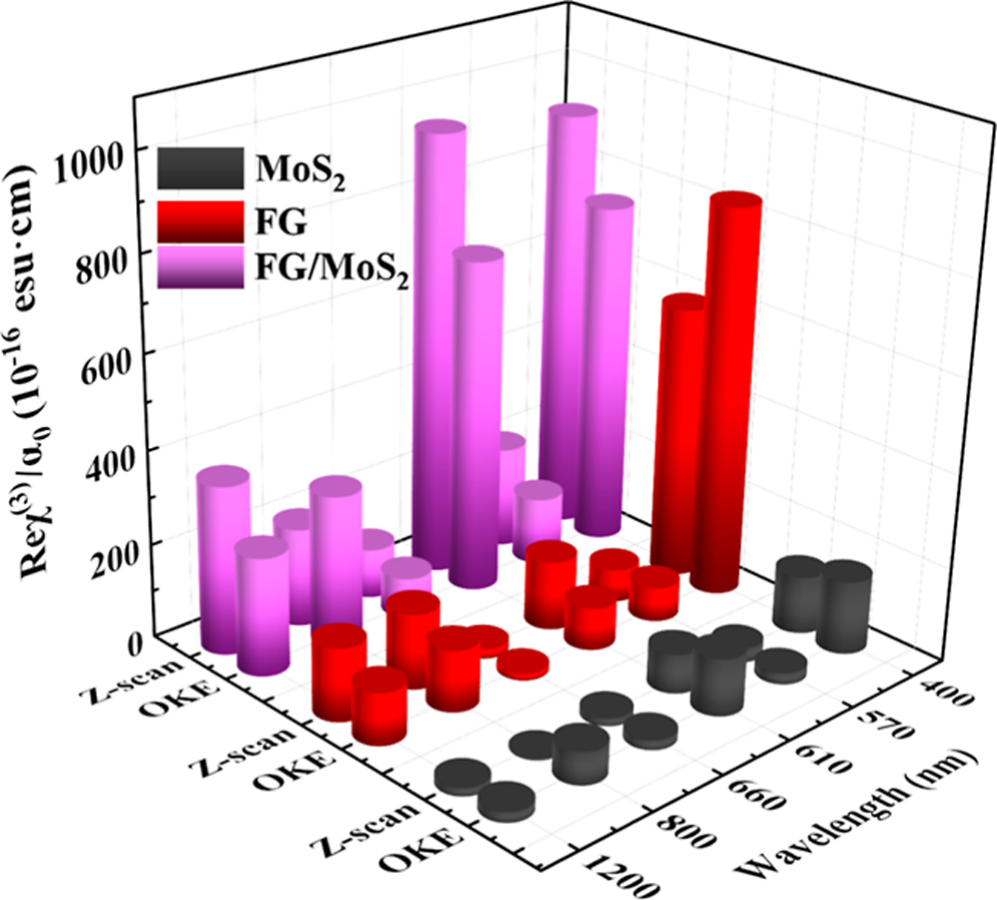
Comparison of the FOM values of Reχ^(3)^ (i.e., |Reχ^(3)^|/α_0_ values) for the studied MoS_2_, FG, and FG/MoS_2_ heterostructure obtained by the *Z*-scan and OKE techniques.

A comparison of the results obtained from the OKE and *Z*-scan techniques is schematically depicted in Figure 10. As can be seen, excellent agreement between the results of both techniques was found. It is worth noting that due to the enhancement of the NLO refraction and Kerr signal of the studied nanostructures for irradiation wavelengths leading to excitation of the excitons of the nanostructures, they hold great promise for serving as all-optical switchers in various fields, including telecommunications, optical computing, data processing, etc.^54^

## Conclusions

In summary, in the present work, the contribution of excitons to the ultrafast NLO properties of MoS_2_ and FG/MoS_2_ heterostructure thin films was evaluated for the first time to the best of our knowledge by performing resonant and off-resonant excitation of their excitons. Under resonant excitation (i.e., with 400, 610, and 660 nm laser radiation), a pronounced enhancement of the NLO response was observed, indicating the significant role of excitons in enhancing the NLO properties of these nanostructures. Due to the excitation of their excitons, the studied MoS_2_ and FG/MoS_2_ heterostructure thin films exhibited exceptional SA and self-defocusing behavior, suggesting their potential for generating ultrashort laser pulses and for ultrafast optical switching applications. The present findings deepen our understanding of the effects of excitons on the NLO properties of TMDs-based nanostructures, highlighting the crucial role of excitons in enhancing the NLO response for advanced applications in photonics and optoelectronics.

## Supporting Information Available

AFM images; optical images; Raman spectra; Raman mapping; methodology for NLO measurements; Tauc plots; energy-dependent Δ*Τ*_p–v_ values; energy-dependent transmittance; SA properties; optical limiting; and stability of the samples during NLO measurements (PDF)
