# Supplementary material for: Excitonic Effects on the Ultrafast Nonlinear Optical Response of MoS2 and Fluorinated Graphene/MoS2 Heterostructure Films for Photonic Applications
Source: ACS Appl Mater Interfaces. 2024 Nov 8;16(46):63951–63. doi: 10.1021/acsami.4c16405 (PMC11583122; doi:10.1021/acsami.4c16405)
Supplement: Supplementary file 1 — am4c16405_si_001.pdf [file am4c16405_si_001.pdf]

Supporting Information

Excitonic Effects on the Ultrafast Nonlinear Optical Response of MoS_2 and Fluorinated Graphene/MoS_2 Heterostructure Films for Photonic Applications

Vasileios Arapakis,[a,b] *Michalis Stavrou,*[a,b] *Georgios Skentzos,*[a,b] *Dipak Maity,*[c] *Tharangattu N. Narayanan,*[c] *Stelios Couris*[a,b]*

[a]Department of Physics, University of Patras, 26504 Patras, Greece.

[b]Institute of Chemical Engineering Sciences (ICE-HT), Foundation for Research and Technology-Hellas (FORTH), Patras, 26504 Patras, Greece

[c]Materials & Interface Engineering Laboratory, Tata Institute of Fundamental Research Hyderabad, Serilingampally Mandal, Hyderabad 500046, India.

Corresponding Author

* Stelios Couris (e-mail: couris@upatras.gr, couris@iceht.forth.gr)

KEYWORDS: MoS_2, Fluorographene, Excitonic Effects, Z-scan, Optical Kerr Effect

AFM images

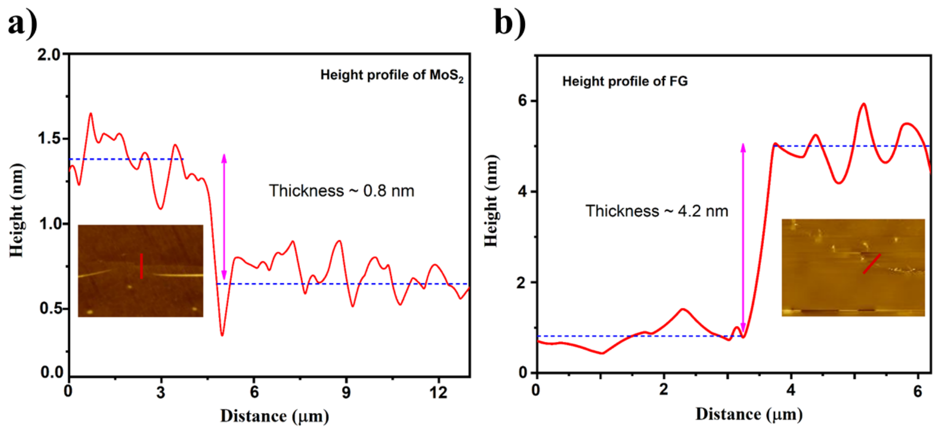

Figure S1. AFM images of (a) MoS_2 and (b) FG.

Optical images

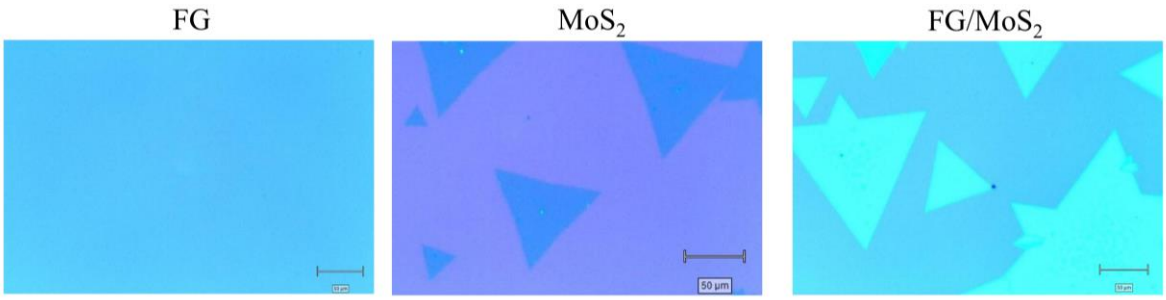

Figure S2. Optical image of FG, MoS_2 and FG/MoS_2.

Raman spectra

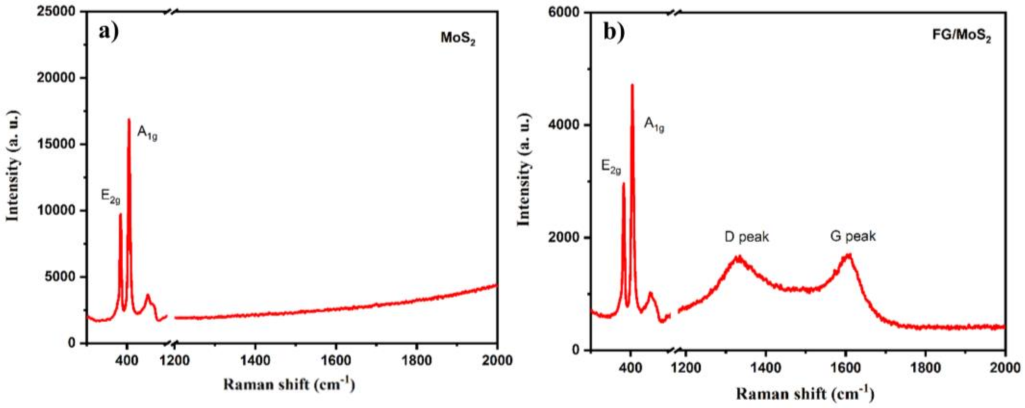

Figure S3. Raman spectra of (a) MoS_2 and (b) FG/MoS_2 over Si/SiO_2 substrate (300 nm).

Raman mapping

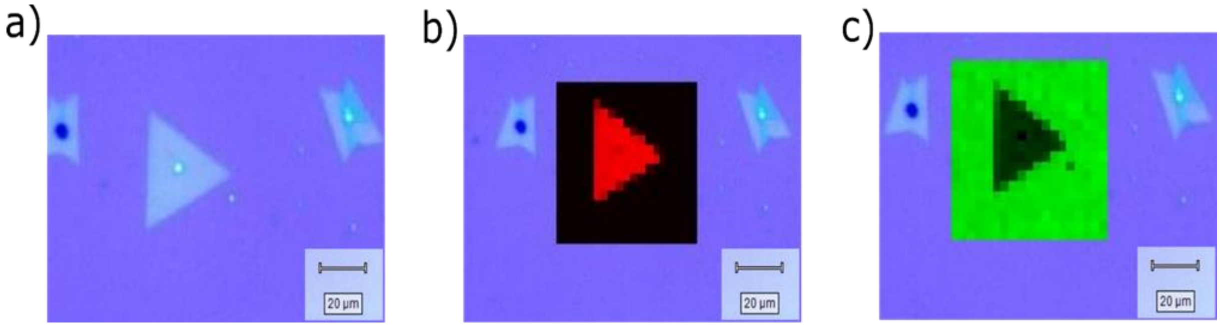

Figure S4. a) optical image of FG/MoS_2 b) mapping of A_{1g} peak of MoS_2 (404 cm^{-1}, red graph) c) mapping of G peak of FG (1600 cm^{-1}, green graph).

Methodology for NLO measurements

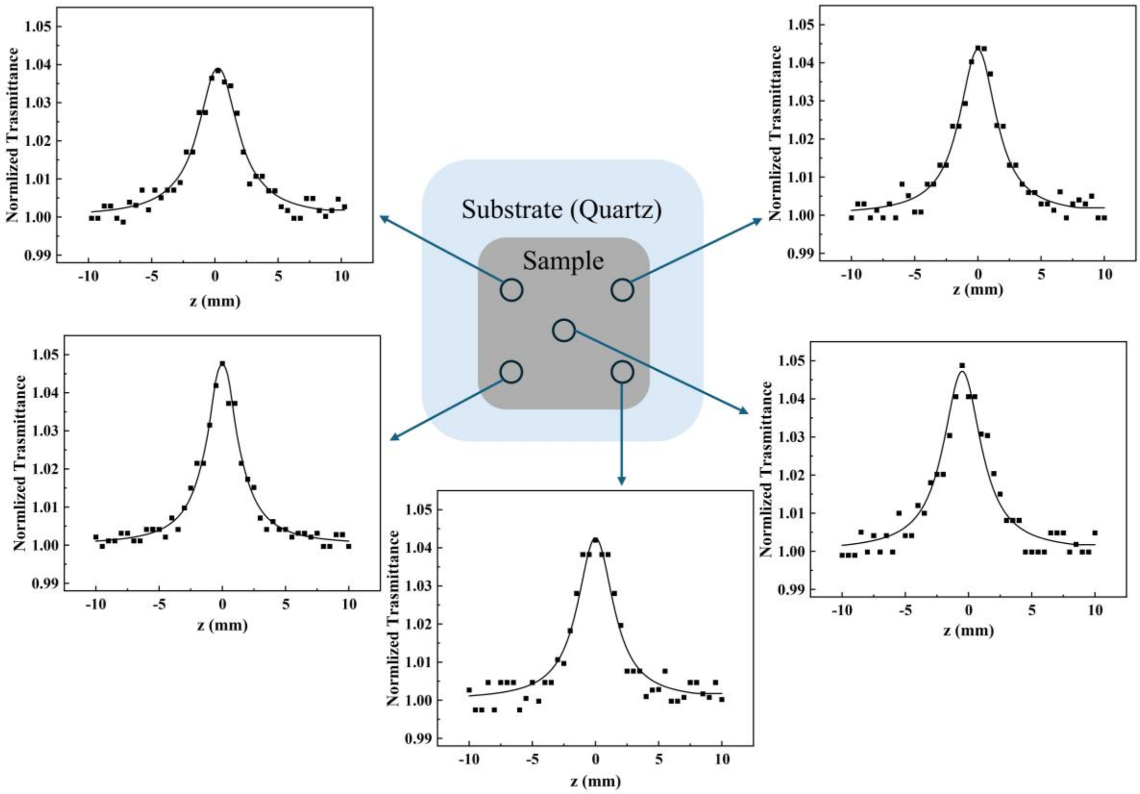

Figure S5. OA Z-scans of FG/MoS_2 at different positions on the surface of the film.

Tauc plots

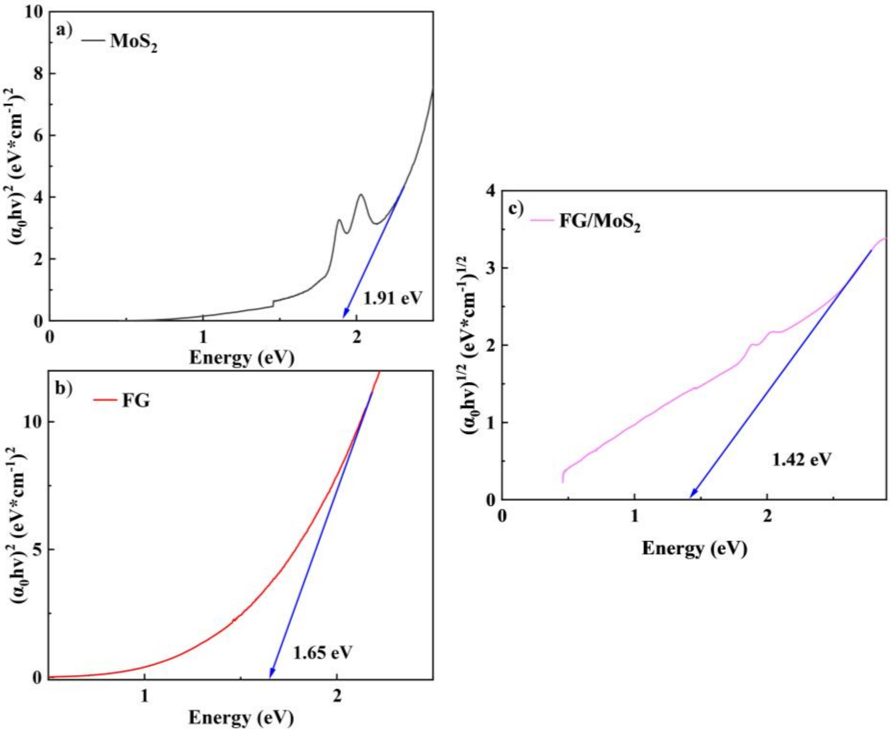

Figure S6. Tauc plot diagrams of (a) MoS_2, (b) FG and (c) FG/MoS_2.

Energy-dependent $\Delta T_{p\text{-}v}$ values

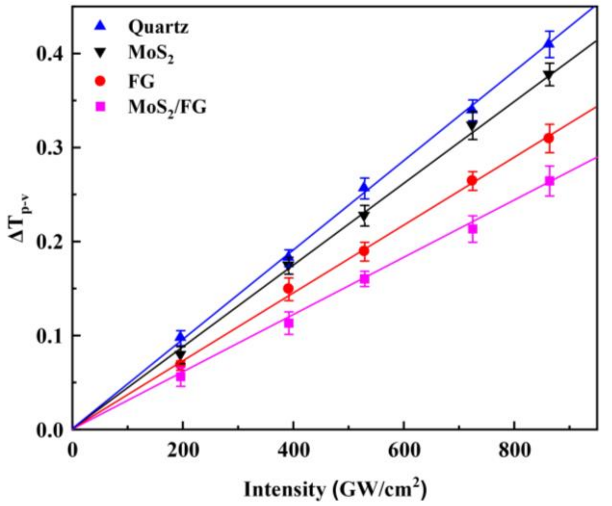

Figure S7. Variation of the $\Delta T_{p\text{-}v}$ values of FG, MoS_2 and FG/MoS_2 as a function of the incident laser intensity (under 800 nm laser excitation).

Energy-dependent transmittance

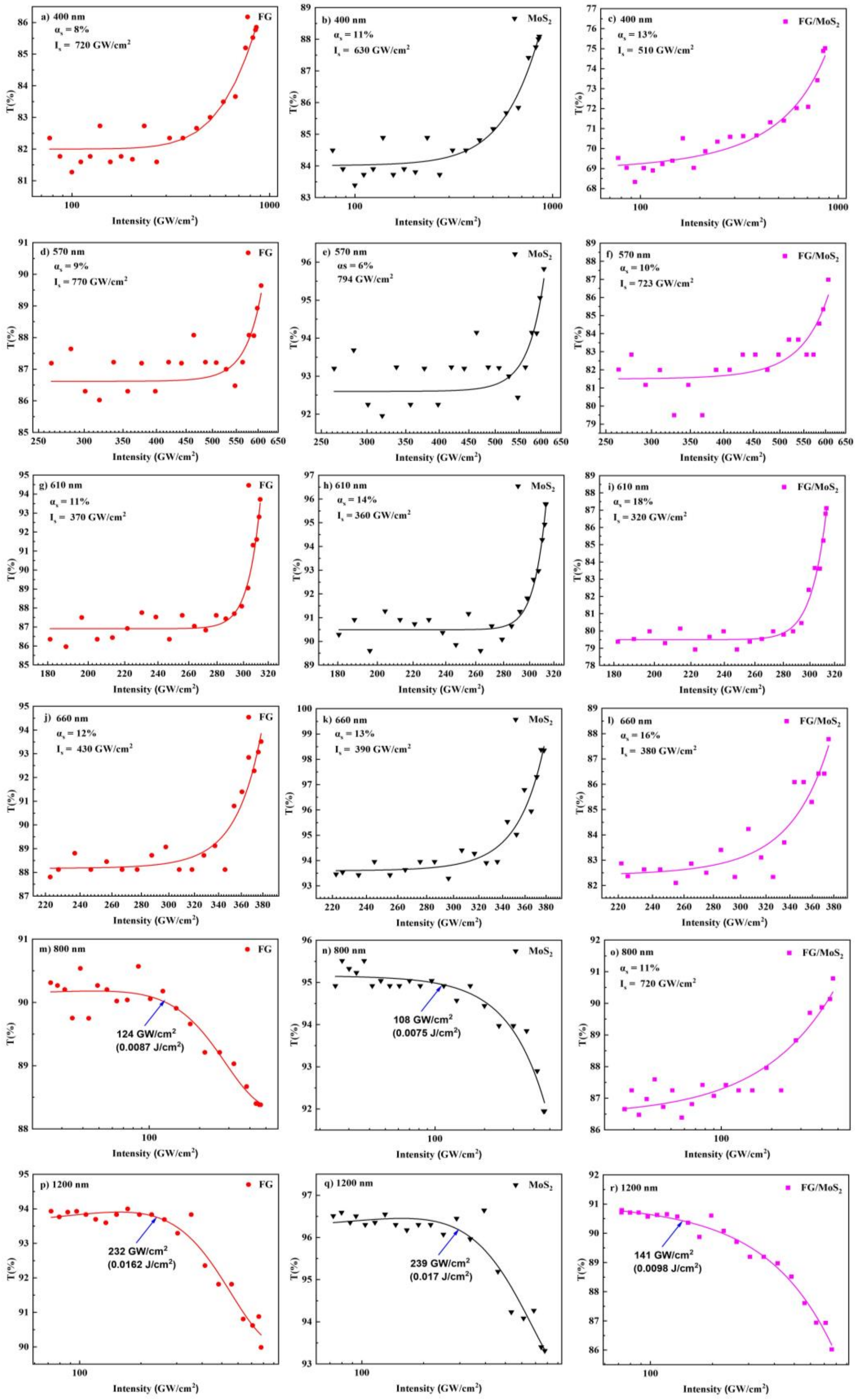

Figure S8. Transmittance of FG, MoS_2 and FG/MoS_2 films under different laser excitation intensities at (a-c) 400, (d-f) 570, (g-i) 610, (j-l) 660, (m-o) 800, and (p-r) 1200 nm.

Saturable absorption properties

According to the typical saturable absorption model,[1] the intensity-dependent transmittance of a sample can be described by the following relation:

$$T = 1 - \left(\frac{\alpha_s}{1+I/I_{sat}} + \alpha_{ns}\right) \quad \text{(S1)}$$

where α_s is the modulation depth, α_{ns} is the non-saturable absorption, I_{sat} is the saturable intensity, and I is the incident laser intensity.

The parameters α_s, α_{ns}, and I_{sat} are obtained by fitting the experimental transmittance shown in Figure S8 with Eq. (1). The results of the fitting showed that the non-saturable absorption is negligible, while the determined values of α_s and I_{sat} are listed in Table S1.

Table S1. Saturable absorption properties (modulation depth and saturable intensity) of FG, MoS_2 and FG/MoS_2 under different excitation wavelengths.

**Sample**	**λ (nm)**	**α_s (%)**	**I_s (GW/cm^2)**
FG	400	8	720
MoS_2		11	630
FG/MoS_2		13	510
FG	570	9	770
MoS_2		6	794
FG/MoS_2		10	723
FG	610	11	370
MoS_2		14	360
FG/MoS_2		18	320
FG	660	12	430
MoS_2		13	390
FG/MoS_2		16	380
FG	800	-	-
MoS_2		-	-
FG/MoS_2		11	720
FG	1200	-	-
MoS_2		-	-
FG/MoS_2		-	-

Optical limiting

The optical limiting efficiency of the films exhibiting RSA behavior under 800 and 1200 nm irradiation was assessed by determining the optical limiting onset (OL_{on}), defined as the incident laser intensity at which the sample's transmittance begins to deviate from the Beer-Lambert regime. As shown in Figures S8(m,n) and S8(p-r), the OL_{on} values of FG and MoS_2 under 800 nm laser pulses were determined to be ~0.0087 and ~0.0075 J/cm², respectively, while under 1200 nm laser irradiation, the OL_{on} values of FG, MoS_2, and FG/MoS_2 were ~0.0162, ~0.017, and ~0.0098 J/cm², respectively.

Stability of the samples during NLO measurements

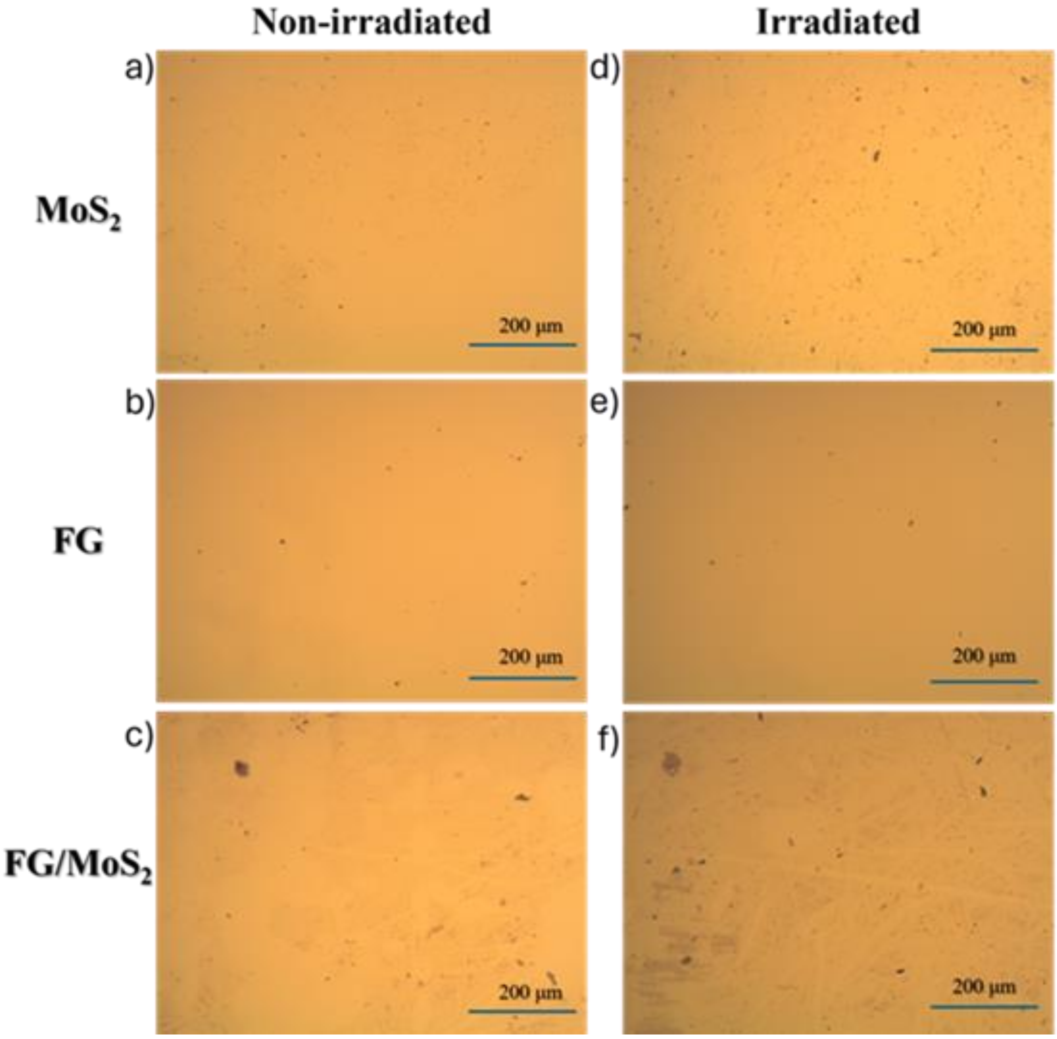

Figure S9. Optical images of FG, MoS_2, and FG/MoS_2: (a-c) before laser irradiation and (d-f) after laser irradiation at different laser intensities.

To ensure the stability of the samples during the NLO measurement, photos of non-irradiated (i.e., not previously used) samples were taken using an optical microscope. Then, Z-scan experiments were performed on these samples, for the range of laser intensities used, and up to 860 GW/cm^2. Between the measurements performed at different laser intensities and up to the maximum laser intensity used, photos of the samples were taken systematically. Some of the obtained photos, before, and after irradiation, are presented in Figure S9. As can be seen from these figures, the irradiated samples do not present any sign(s) of creation of holes or other visible signs of laser ablation.

Moreover, several Z-scan measurements were performed consecutively at the same position of the films, and at the largest laser intensity used for the NLO measurements (i.e., ~860 GW/cm^2) in order to check if any variation of the transmittance of the MoS_2, FG and FG/MoS_2 samples was occurring during the Z-scan experiments. As can see from the "Open-aperture" Z-scans of FG/MoS_2 presented in Figure S10, the measured transmittance of the samples remained unchanged after four consecutive scans at the same point.

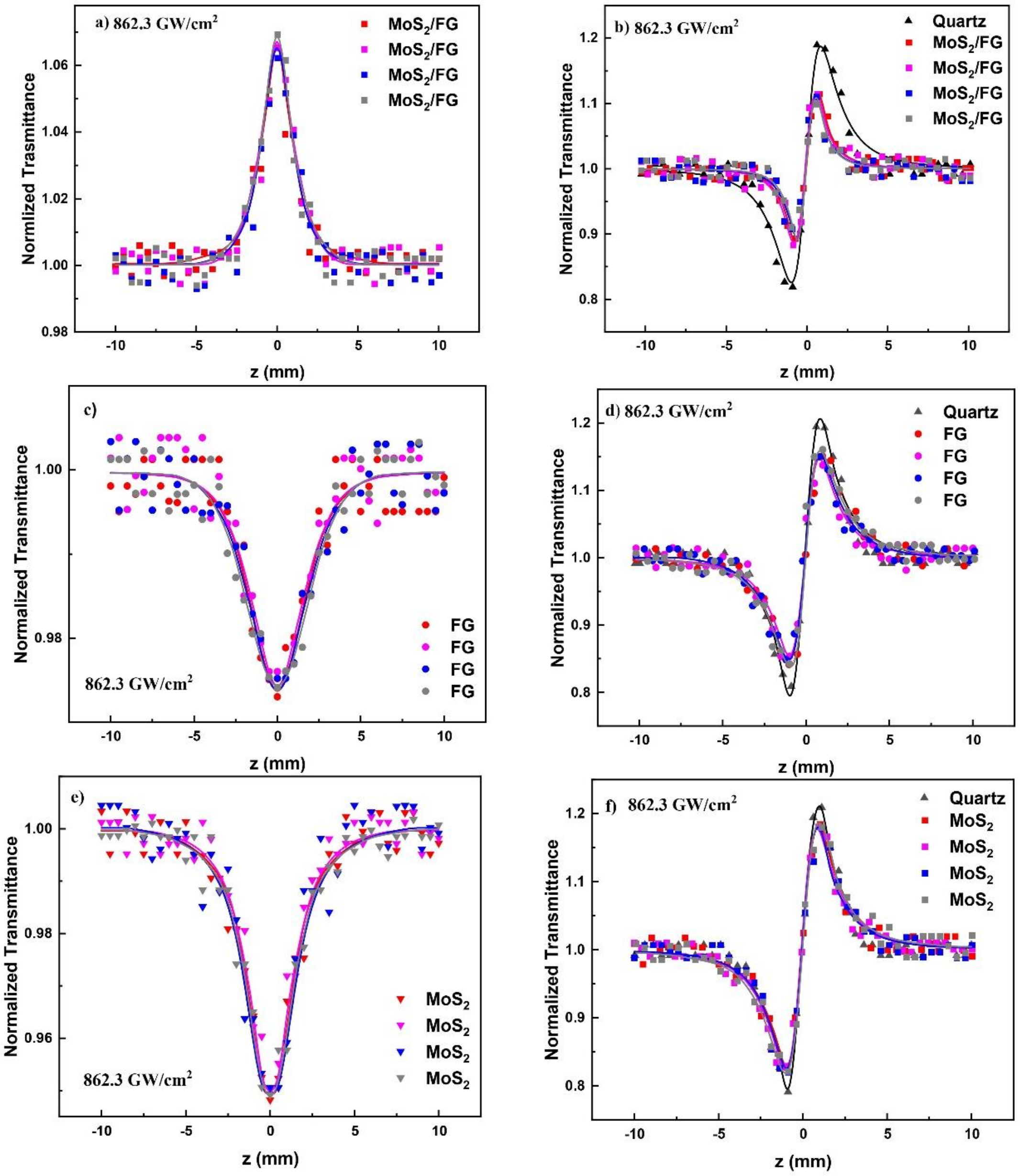

Figure S10. (a, c, e) "Open-aperture" and (b, d, f) "Closed-aperture" Z-scans at the same position of FG, MoS_2, and FG/MoS_2 films under 70 fs, 800 nm laser excitation, all corresponding to the same laser intensity of ~860 GW/cm².

Similarly, in Figure S7 the measured $\Delta T_{p\text{-}v}$ values (i.e., the difference of the normalized transmittance between the peak and the valley of the "divided" Z-scan) as a function of the laser intensity are depicted. As shown, they were all found to scale linearly with the laser intensity, for all films. From the slopes of these straight lines the nonlinear refractive index

parameter γ' is calculated. This result again confirms the stability of the films during the measurements.

References

(S1) Garmire, E. Resonant Optical Nonlinearities in Semiconductors. *IEEE J. Sel. Top. Quantum Electron.* **2000**, *6*, 1094–1110.
